# Gestational Diabetes In Rural Women of Jammu

**DOI:** 10.4103/0970-0218.39247

**Published:** 2008-01

**Authors:** AK Verma, Bhupinder Singh, Vijay Mengi

**Affiliations:** Post Graduate Department of Community Medicine, Government Medical College, Jammu, J and K, India

## Introduction

Based on demographic projections made by United Nations Population Division for the year 2025, WHO issued estimates of adults with diabetes in all countries and reported that there will be more women with diabetes than men and we may anticipate a considerable increase in the burden of gestational diabetes mellitus (GDM) especially in less prosperous countries.(1) During last two decades there has been a marked increase in the prevalence of diabetes among urban Indian.(2) Similar, though slower trend is also shown amongst peri-urban and rural population due to changes in life style, ageing and low birth weight leading to diabetes during adulthood.(3) This epidemic has been triggered by social and economic development and urbanization associated with general improvement in nutrition, longevity and reduced physical activity. Present study was taken up to study the influence of the socio-economic status of the women and gestational diabetes in a rural area.

## Materials and Methods

A rural health block under the Government Medical College was selected randomly. Block comprised of 8 zones and 24 sub centers for health care deliverance. Out of 200 Anganwadi centers (AWCs) 9 were excluded as they catered to urban populace. Giving due representation to all the 8 zones of the block, 100 AWCs were selected randomly and all the expectant mothers in the respective AWC were registered for the study. The group was interviewed, physically examined and screened according to pre- structured and pre-tested proformas. Every woman was contacted thrice for the purpose consecutively for three days. For assessing the level of activity, subjects were categorized as sedentary, moderate workers and heavy workers according to recommendation of WHO expert committee. Urinalysis was done for sugar by URS-IG uristix strips(urine reagent strips for the semiquantative and qualitative detection of glucose). On the first day of contact the subject was asked to remain fasting over night. Next day, subject was asked to drink 75 grams of glucose in 200 ¨C 300 ml of water in 5-7 minutes. Capillary whole blood sugar (CWBS) was estimated after two hours of OGTT as per WHO guidelines using Accu-Chek-Glucometer.

## Results

455 women were registered in all. 11 having thyromegaly were excluded. 38 could not be contacted or did not consent for OGTT leaving 406 as study population. The mean CWBS was 103.7 ± 22.8 mg/dl (95% CI 101.01-106.30). 380 subjects were normoglycemic, while 26 had blood sugar between ¡Ý140 ¡Ü 200 mg/dl. For risk analysis of age, the subjects were grouped into¡Ü 25 or ¡Ý25 years of age. The association of age and diabetes was found to be statistically non significant [Table 1]. 61.58% of our population was matriculate or had received higher education. However, level of literacy showed no bearing on the glycemic state of the expectant mothers. Though, the main occupation of study area was agriculture [Table 2], 358 (88.18%) of mothers were housewives, maximum engaged in moderate physical work [Table 3]. The occupation was the only variable in this study which had the significant statististical influence on the diabetic state of the mother. Maximum 76.3% (318) women had income in the lowest category but the association between various income groups and glycemic state was statistically non significant (*P* >0.05).

**Table 1:** Age and distribution of diabetes mellitus in pregnancy

Blood sugar (mg/dl)	<25 yrs (n) (%)	Age in years ¡Ý 25 yrs (n) (%)	Group total (n) (%)
¡Ü140	167 (43.94)	213 (56.06)	380 (100)
¡Ý140¡Ü200	14 (53.84)	12 (46.16)	26 (100)
Total	181 (44.58)	225 (55.42)	406 (100)

χ^2^ = 0.967 df 1 *P*.32 Non-significant

**Table 2:** Occupation and distribution of diabetes in pregnancy

Blood sugar (mg/dl)	Occupation					
	
	Laborer (n) (%)	Professional (n) (%)	Service (n) (%)	Business (n) (%)	Housewife (n) (%)	Total (n) (%)
¡Ü140	41 (10.79)	0	4 (1.05)	1 (0.26)	334 (87.90)	380 (100)
¡Ý140¡Ü200	1 (3.84)	1 (3.84)	0	0	24 (92.32)	26 (100)
Total	42 (10.34)	1 (3.84)	4 (0.98)	1 (0.25)	358 (88.18)	406 (100)

χ^2^ = 16.15 *P* 0.002 Highly significant

**Table 3:** Physical activity and distribution of gestational diabetes

Blood sugar (mg/dl)	Physical activity			
	
	Mild (n) (%)	Moderate (n) (%)	Heavy (n) (%)	Total (n) (%)
¡Ü140	5 (1.32)	346 (91.05)	29 (7.63)	380 (100)
¡Ý140¡Ü200	0	25 (96.16)	1 (3.84)	26 (100)
Total	5 (1. 2)	371 (91.4)	30 (7.4)	406 (100)

χ^2^ = 0.883, *P* 0.643 Non-significant

## Discussion

Many studies have been conducted on gestational diabetes in urban areas to find out prevalence using different methods of diagnosis but in glaring contrast there is paucity of work in rural areas. Worldwide prevalence of GDM varies between 0.6-13.7% (WHO) criteria.(4) One such study conducted in rural area s of Ethiopia shows prevalence of GDM to be at 3.7%.(5) In the present study the prevalence rate was 6.7%. This may be attributable to use of WHO criteria, universal screening and ethnic difference from south Indian population and presence of hostilities at this place (Indo-Pak border).

The mean age of study group was 25.2 ± 7.6 years. Various authors from India have observed GDM in higher age groups, majority of which were carried out in urban areas. Sikdar *et al.*,(6) reported GDM in age group 31-35 years. The study conducted by Seyoum *et al.*,(5) in 18 rural villages of Northern Ethiopia reported GDM in mothers having mean age of 27.4 ± 7.1 which is in accordance with our study.

Majority of our study population was matriculate or had received higher education. It was observed that an increase in level of literacy increased the prevalence of GDM but it was not statistically significant, thus indicating no influence of literacy on GDM.

## Conclusion

There is moderate prevalence of GDM in this study. We also observed the changed life style of the rural population. It necessitates the need of close monitoring of the expectant mothers for diabetes by the health care system.
